# Biochemical Changes in Prostate Cancer: FMNL1 and PAK1 in Plasma and Urine

**DOI:** 10.3390/cimb47080648

**Published:** 2025-08-13

**Authors:** Elif Bilgin Doğru, Selçuk Erdem, Hilal Oğuz Soydinç, Ayça İribaş, Derya Duranyıldız

**Affiliations:** 1 Department of Basic Oncology, Institute of Oncology, Istanbul University, 34093 Istanbul, Turkey; hoguz@istanbul.edu.tr (H.O.S.); deryady@istanbul.edu.tr (D.D.); 2 Division of Urologic Oncology, Department of Urology, Istanbul Faculty of Medicine, Istanbul University, 34093 Istanbul, Turkey; erdemsel@istanbul.edu.tr; 3 Department of Radiation Oncology, Institute of Oncology, Istanbul University, 34093 Istanbul, Turkey; ayca.iribas@istanbul.edu.tr

**Keywords:** prostate cancer, formin, FMNL1, p21-activated kinase, PAK1

## Abstract

Prostate cancer is a clinically heterogeneous disease. Since PSA is not cancer-specific, and due to the bone metastases seen in the advanced stage and bone deformations caused by hormone therapy, it is necessary to use new biomarkers. Formin-like-protein 1 (FMNL1), a member of the formin protein family, is of great importance in actin polymerization, cell attachment, and migration processes. p21-activated kinase 1 (PAK1) proteins, members of the PAK protein kinases, play a role in cytoskeletal organization, as well as regulating other cellular activities such as cell survival, mitosis, and transcription. In our study, plasma and urine samples of 60 prostate cancer patients and 20 healthy controls were studied using RT-PCR and ELISA methods. No statistical difference was found between FMNL1 mRNA and protein expression levels of patients and controls in both plasma and urine samples (*p* > 0.05). There was no statistical difference between PAK1 mRNA expression levels of patients and controls in plasma and urine samples (*p* > 0.05). While no significant difference was found in PAK1 protein levels in plasma samples (*p* > 0.05), it was found to be lower in urine samples of patients compared to the control group (*p* = 0.00). Both marker molecules have low expression levels in early-stage PCa.

## 1. Introduction

Prostate cancer (PCa) is a clinically very heterogeneous disease in which both genetic and environmental factors play a role in its etiology. PCa, which is the most common cancer type in men in Western societies, occurs with an average of 900,000 new cases every year in the world [1]. Due to the lack of specific markers, widespread bone metastases seen in advanced stages, and bone deformations caused by hormone therapy, it is necessary to use new biomarkers that are both specific for PCa and will provide diagnosis at an early stage.

Formins are multi-domain polypeptides that function by forming homodimers. They take part in the elongation phase of the actin polymerization process. Some formins can also initiate the formation of new actin filaments from G-actin. They control actin homeostasis and cell motility through profilin [2].

FMNL1, expressed in leukocytes, spleen, and thymus, regulates the formation of F-actin networks and the bundling of actin filaments [3,4,5]. It also plays a role in many immunological processes by helping macrophages acquire phagocytosis, adhesion, and migration abilities by creating actin-rich adhesion units called podosomes [6].

FMNL1 has been shown to be effective in epithelial to mesenchymal transition (EMT) in association with many genes such as VEGF, N-cadherin, Vimentin, VCAM, MMP2, MMP13, and interleukin-8. It has also been determined that FMNL1 epigenetically facilitates EMT by upregulation of metastasis-associated protein 1 [7].

The main functions of the PAK family of serine/threonine kinases are to contribute to cell regulation by affecting cell shape, motility, and adhesion. PAKs control the cytoskeleton by regulating the polymerized actin structures, especially the formation of filopodia and lamellipodia [8].

It is known that PAK1 is overexpressed in almost all types of cancer. It was first determined that PAK1 was effective in the MAPK pathway, and then it was reported that it played a role in actin skeleton formation. In the 20 years since it was found to be effective in the proliferation and progression of breast cancer, the role of PAK1 in cancer formation continues to be investigated [9,10].

In addition to its functions affecting oncogenic transformation, such as facilitating the adaptation of tumor cells to a hypoxic environment by activating HIFs, supporting angiogenesis by interacting with bFGF and VEGF, and helping tumor cells escape from the immune system and apoptosis, PAK1 has a role in many signaling pathways [11,12,13,14,15].

PAK1 promotes EMT and metastasis through multiple pathways. The degradation of the adhesive connections between epithelial cells is carried out by many EMT transcription factors. All of these transcription factors can also be activated by PAK1. Thus, cancer cells acquire even more metastatic properties through a signaling network regulated by PAK1 [16,17].

Given the significant roles of FMNL1 and PAK1—two molecules involved in actin polymerization and cell motility—we hypothesized that their expression levels would be elevated in prostate cancer patients, particularly in advanced stages. To date, no studies have investigated the combined activity of these two molecules. Moreover, the absence of research examining their individual or combined levels in patients’ blood and urine samples, as well as the potential association between these levels and disease progression, motivated us to conduct this study.

## 2. Materials and Methods

### 2.1. Patients

In total, 60 (male) PCa patients admitted to Istanbul University, Institute of Oncology, and Istanbul Medicinal Faculty, Department of Urology between 2016 and 2018 were enrolled in the study. Of 60 PCa patients, 38 were determined to be local, 18 locally advanced, and 4 metastatic. The average age of PCa patients was determined as 65.7 (46–78) years. Characteristics of PCa patients are given in Table 1.

Blood and urine samples were collected during the initial admission, prior to the administration of any treatments such as chemotherapy, radiotherapy, hormone therapy, or targeted therapy. All patients and healthy controls provided written informed consent for sample collection and testing. The patient samples were then centrifuged (using a Hettich Universal 32) at 4000 rpm for 10 min. The separated samples were preserved at −80 °C until further analysis. The pretreatment assessment involved a comprehensive clinical history, a physical examination, and a series of biochemical tests, including the measurement of serum PSA levels.

For comparison of FMNL1 and PAK1 levels, age-matched 20 healthy (male) controls were included in the analysis.

### 2.2. Measurement of Plasma/Urine mRNA Expression Levels of FMNL1 and PAK1

#### 2.2.1. RNA Isolation

An amount of 1 mL of the sample was transferred into tubes, followed by the addition of 2 mL RNAzol BD (Molecular Research Center, Inc., Ohio, OH, USA), 10 μL glacial acetic acid, and 1 μL Polyacryl Carrier (Molecular Research Center, Inc., Ohio, OH, USA). The mixture was shaken for 15–30 s. Then, 150 μL of 1-bromo-3-chloropropane (Molecular Research Center, Inc., Ohio, USA) was added to the lysate. After incubation and centrifugation, isopropanol was added to the supernatant. After another incubation and centrifugation step, the liquid was removed, and 75% ethanol was added to the pellet at the bottom, which was then mixed. The RNA pellet was resuspended in RNase-free water without drying. The concentrations of the isolated RNAs were then measured.

#### 2.2.2. Complementary DNA (cDNA) Synthesis

cDNA synthesis was performed using a commercial cDNA kit (Jena Bioscience GmbH, Jena, Germany) with the total RNAs obtained. RNA concentrations were standardized before initiating the cDNA synthesis process. The reaction was performed for each sample as described in Table 2, with a final volume of 20 μL. The sample mixtures were placed in a plate and subjected to a conventional PCR device (Biorad CFX Connect, Bio-Rad Laboratories, Inc., California, USA). cDNA synthesis was completed through incubation at 42 °C for 10 min, followed by 50 °C for 60 min, and 70 °C for 10 min. The resulting cDNA samples were stored at −20 °C.

#### 2.2.3. Real-Time Polymerase Chain Reaction (RT-PCR)

Following cDNA synthesis, mRNA expression levels of FMNL1 and PAK1 genes were assessed using real-time polymerase chain reaction (RT-PCR) on a LightCycler 480 (Roche Diagnostics, Basel, Switzerland) system with the SYBR Green Master PCR Kit (Jena Bioscience GmbH, Jena, Germany, Cat No: PCR-372). The β-actin gene was employed as the reference gene.

All procedures were conducted on ice, with a reaction volume of 20 μL. The reaction mixture was prepared by combining 0.6 μL of forward primer, 0.6 μL of reverse primer, 6.8 μL of PCR-grade water, 10 μL of qPCR SybrMasterMix, and 2 μL of cDNA. The prepared sample mixtures were placed in a 96-well plate and subjected to the RT-PCR process. The reaction began with an initial denaturation step at 95°C for 2 min, followed by 40 cycles consisting of denaturation at 95 °C for 15 s, annealing at 52 °C for 1 min, and extension at 60 °C for 1 min. The gene sequences for the synthesized primers are provided in Table 3.

The amplification of the target product for each molecule was verified through melting curve analysis. The expression levels of each RNA are presented as threshold cycle (Ct) values, which represent the fractional cycle number at which the fluorescent signal surpasses a predefined threshold in qRT-PCR. Data analysis was carried out by averaging the Ct values obtained from two independent replicate experiments. In this comparative Ct method, the values were normalized by subtracting the Ct value of the endogenous reference gene from the Ct value of each RNA.

### 2.3. Measurement of Plasma/Urine Protein Levels of FMNL1 and PAK1

The protein levels of FMNL1 and PAK1 (Abbkine Scientific Co., Ltd., Wuhan, China) were measured using the solid-phase sandwich ELISA technique. The microtiter plate was pre-coated with an antibody specific to FMNL1/PAK1. Standards and samples were added to the wells along with a biotin-conjugated antibody that specifically binds to FMNL1/PAK1. Avidin conjugated to horseradish peroxidase (HRP) was then added to each well and incubated. Following the addition of tetramethylbenzidine (TMB) substrate solution, a color change occurred in the wells containing FMNL1/PAK1, the biotin-conjugated antibody, and the enzyme-conjugated avidin. The enzyme–substrate reaction was stopped by adding a sulfuric acid solution, and the color change was measured spectrophotometrically using a ChroMate^®^4300 (Awareness Technology, Inc., Palm City, FL, USA) at a wavelength of 450 nm. The concentration of FMNL1/PAK1 in the samples was calculated by comparing the optical density (OD) of the samples to the standard curve.

### 2.4. Statistical Analysis

Statistical analyses were conducted using SPSS Software (SPSS 16, Chicago, IL, USA). After performing normality tests on the variables with the Kolmogorov–Smirnov and Shapiro–Wilk methods, the non-parametric Mann–Whitney U test was selected to assess statistical significance between the parameters. A *p*-value of less than 0.05 was considered statistically significant. Correlation coefficients and their significance were determined using the Spearman test. The diagnostic performance of the tests was evaluated using the ROC curve.

## 3. Results

### 3.1. mRNA Expression Levels

The arithmetic mean, standard deviation, and median values of plasma FMNL1 mRNA expression levels were found to be 1.33 ± 1.96, 0.66 and 1.64 ± 1.61, 1.44 in PCa patients and healthy controls, respectively. Arithmetic mean, standard deviation and median values of urinary FMNL1 mRNA expression levels in patients were 1.93 ± 6.58, 0.42, and it was found to be 1.06 ± 1.61, 1.44 in controls. The arithmetic mean, standard deviation, and median values of plasma PAK1 mRNA expression levels were found to be 0.70 ± 1.37, 0.15 and 0.96 ± 1.52, 0.25 in patients and controls, respectively. The arithmetic mean, standard deviation, and median values of urinary PAK1 mRNA expression levels were found to be 1.07 ± 2.16, 0.39 in patients and 0.93 ± 0.66, 0.83 in controls (Table 4).

Although plasma and urine FMNL1 mRNA expression levels in PCa patients were found to be lower than in the healthy control group, no statistical significance was found (*p* = 0.182, *p* = 0.126). Plasma and urine PAK1 mRNA expression levels were found to be lower in the patient group than in the control group, but no statistical significance was found (*p* = 0.191, *p* = 0.071).

No statistical significance was found between the age, stage, and PSA values of the patients and the tests (*p* > 0.05).

**Figure 1:**
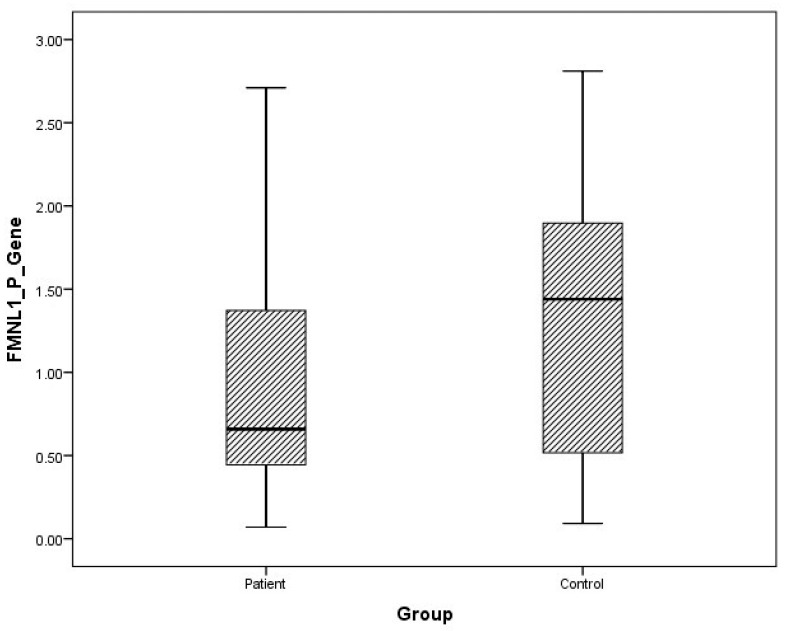
Distribution of mRNA expressions of plasma FMNL1 in patients vs. controls.

**Figure 2:**
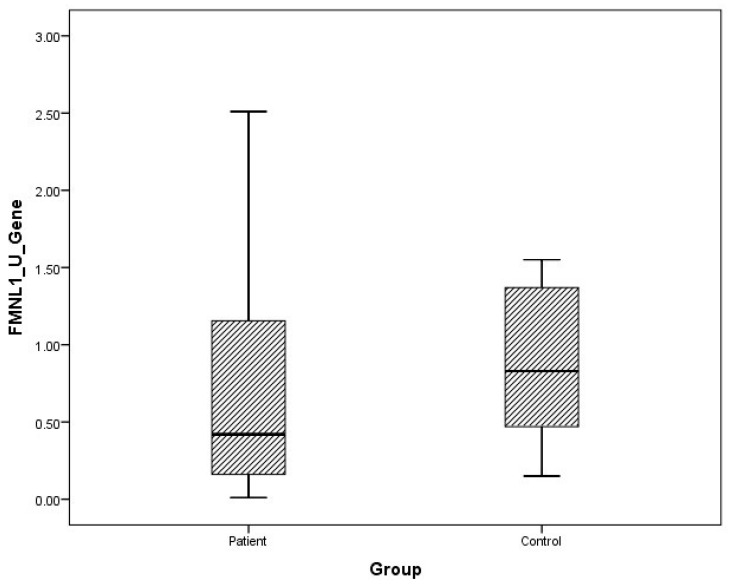
Distribution of mRNA expressions of urine FMNL1 in patients vs. controls.

**Figure 3:**
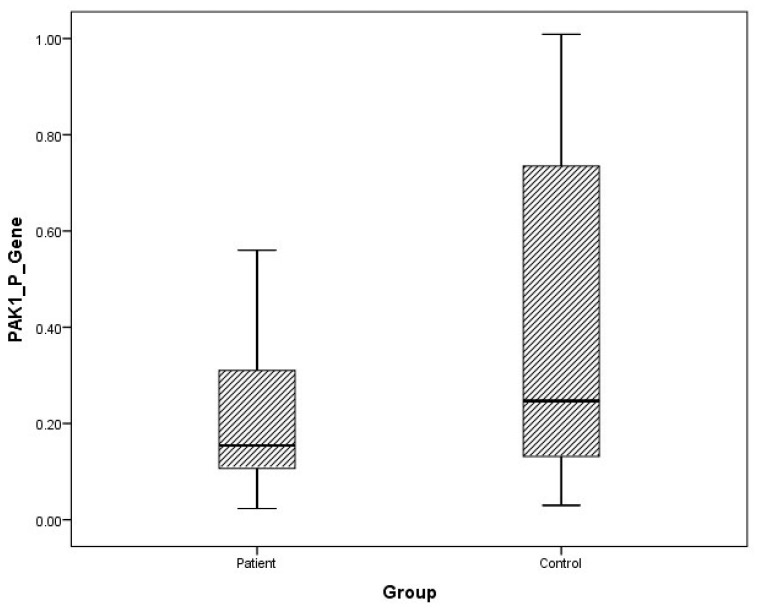
Distribution of mRNA expressions of plasma PAK1 in patients vs. controls.

**Figure 4:**
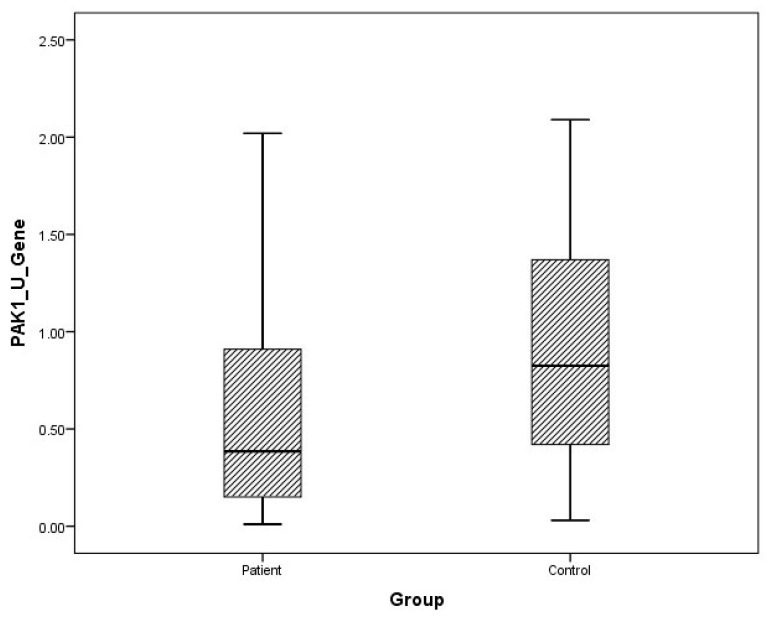
Distribution of mRNA expressions of urine PAK1 in patients vs. controls.

The distribution of mRNA expression levels in patient and control groups is given in the graphs (Figure 1, Figure 2, Figure 3 and Figure 4).

**Figure 5:**
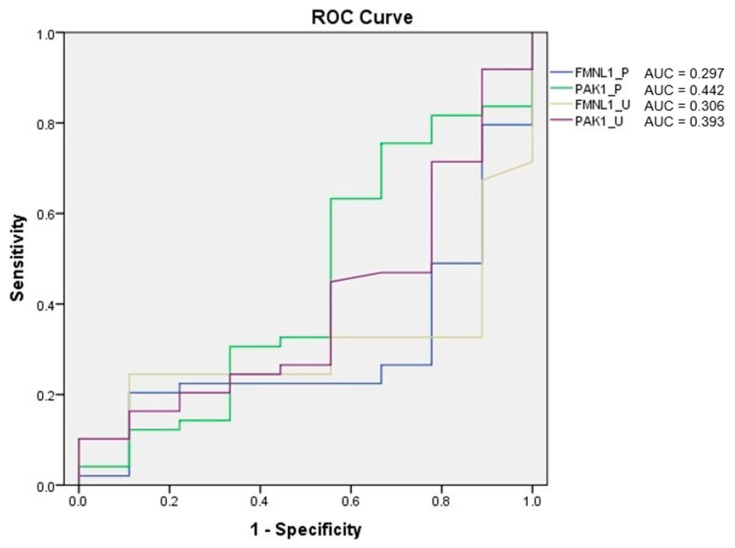
ROC analysis of mRNA expression tests.

Receiver operating characteristic (ROC) analysis of the tests was performed, and the area under curve (AUC) was calculated according to the ROC curves. As a result of the evaluation, it was determined that the diagnostic value of the tests was low (Figure 5).

**Figure 6:**
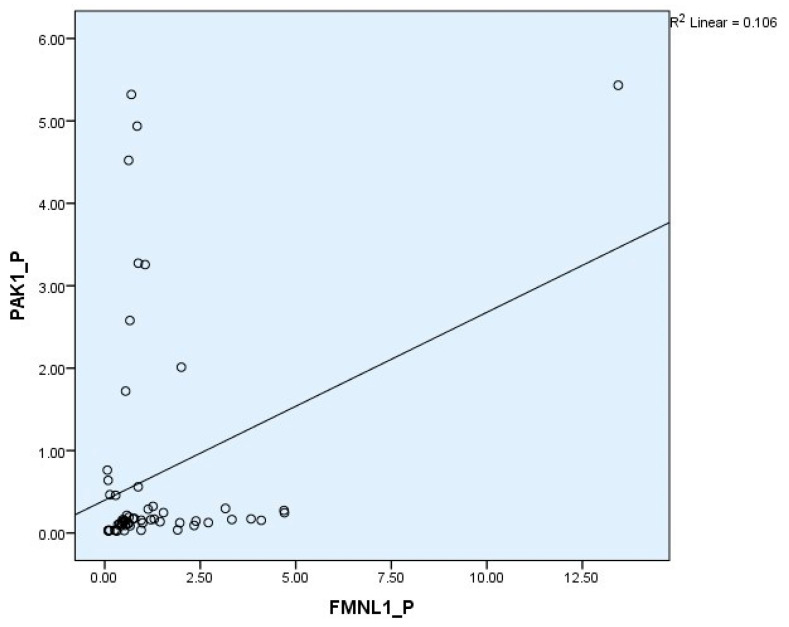
Correlations of plasma mRNA expressions.

**Figure 7:**
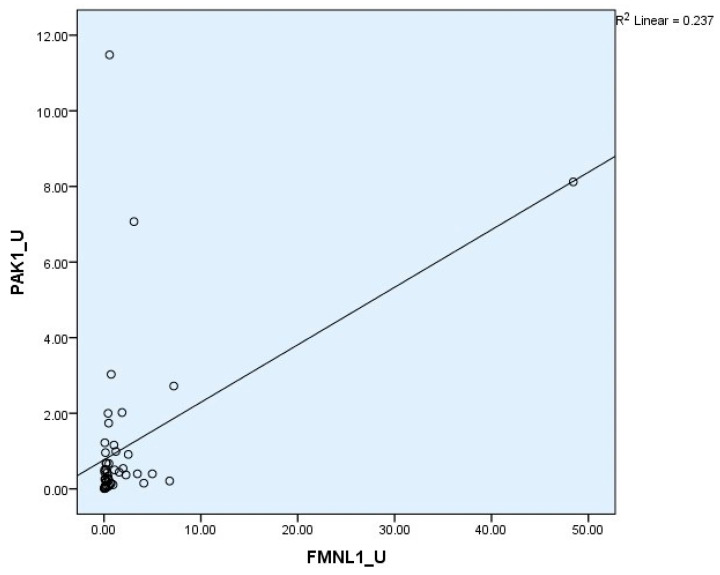
Correlations of urine mRNA expressions.

Correlation between tests was determined with the Spearman test. There was a correlation between plasma FMNL1 and plasma PAK1 mRNA expression levels (*p* = 0.005, r = 0.354) (Figure 6 and Figure 7).

### 3.2. Protein Levels

Arithmetic mean (x), standard deviation (sd), and median (m) values of plasma protein FMNL1 were found to be 425.9 ± 460.2, 357.5 ng/L and 389.7 ± 68.4, 379.8 ng/L in PCa patients and healthy controls, respectively. Arithmetic mean, standard deviation, and median values of urine protein FMNL1 in patients were 509.5 ± 47.8, 508.5 ng/L; it was found to be 520.7 ± 80.3, 544.8 ng/L in controls. The arithmetic mean, standard deviation, and median values of plasma protein PAK1 were found to be 682.5 ± 504.4, 631.8 ng/L and 683.3 ± 101.7, 658.5 ng/L in patients and controls, respectively. Arithmetic mean, standard deviation, and median values of urine protein PAK1 in patients were 614.3 ± 201.1, 669.5 ng/L; it was found to be 868.8 ± 117.2, 893.5 ng/L in controls (Table 5).

Although plasma and urine protein FMNL1 levels in PCa patients were lower than in the healthy control group, no statistically significant difference was detected (*p* = 0.077, *p* = 0.068). No statistically significant difference was detected between plasma protein PAK1 levels in the PCa patient group and the healthy control group (*p* = 0.089). Urinary protein PAK1 levels in PCa patients were found to be lower than in the healthy control group, and a statistically significant difference was detected (*p* = 0.00).

No statistical significance was found between the age, stage, and PSA values of the patients and the tests (*p* > 0.05).

**Figure 8:**
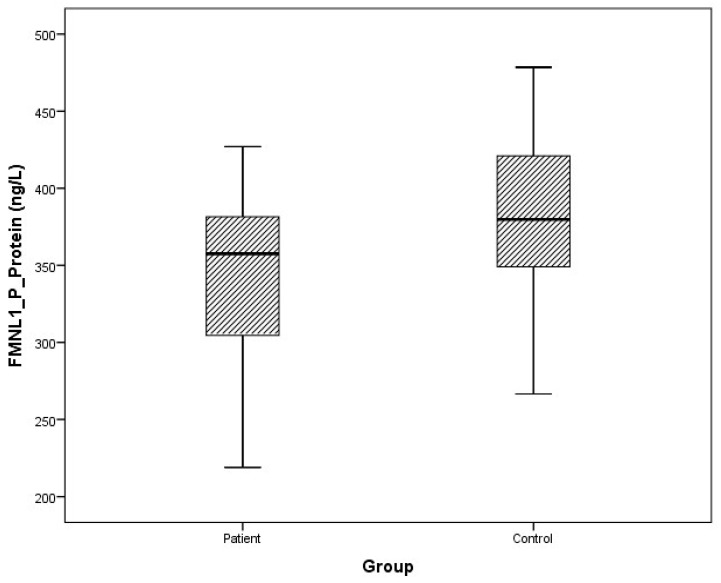
Distribution of protein expressions of plasma FMNL1 in patients vs. controls.

**Figure 9:**
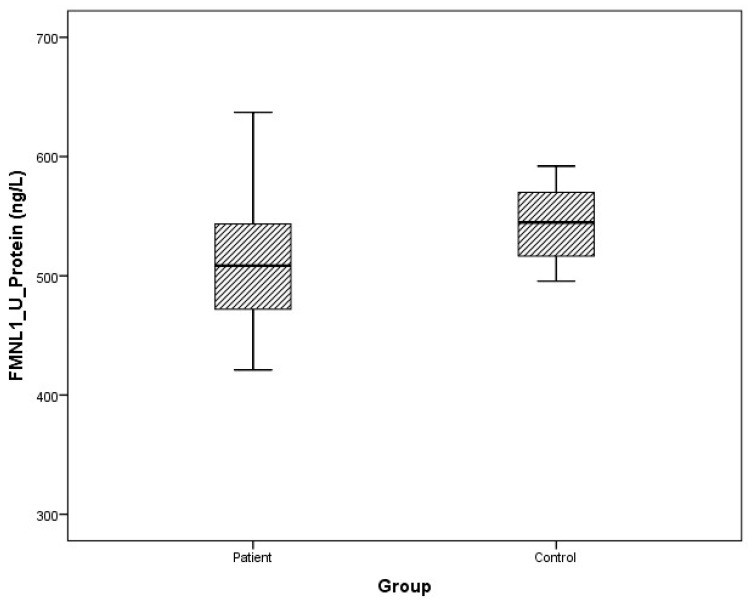
Distribution of protein expressions of urine FMNL1 in patients vs. controls.

**Figure 10:**
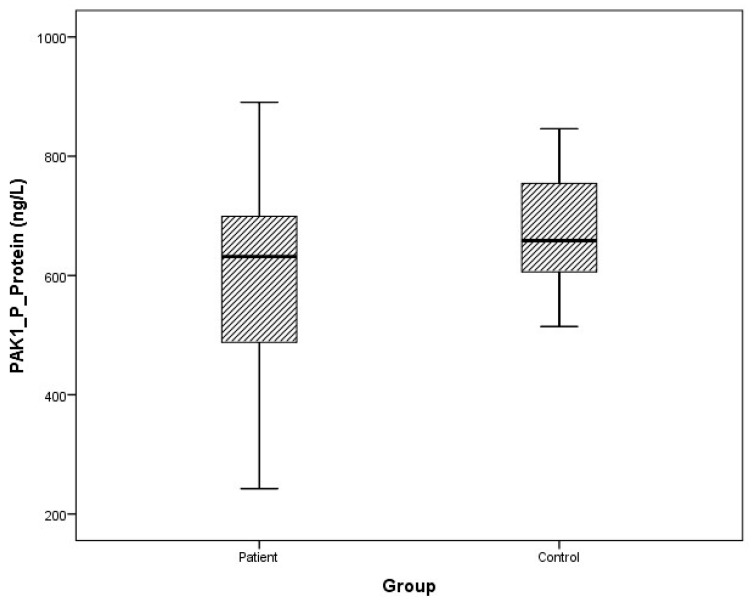
Distribution of protein expressions of plasma PAK1 in patients vs. controls.

**Figure 11:**
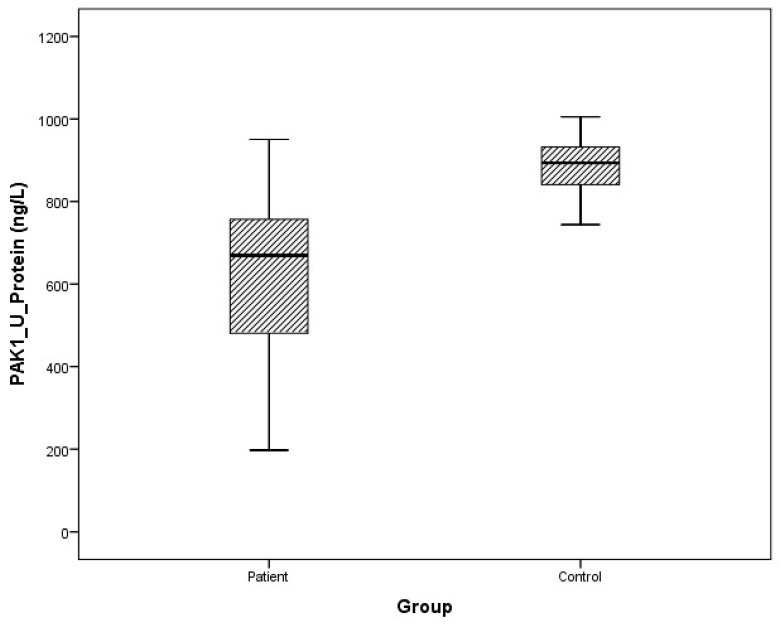
Distribution of protein expressions of urine PAK1 in patients vs. controls.

The distribution of mRNA expression levels in the patient and control groups is given in the graphs (Figure 8, Figure 9, Figure 10 and Figure 11).

**Figure 12:**
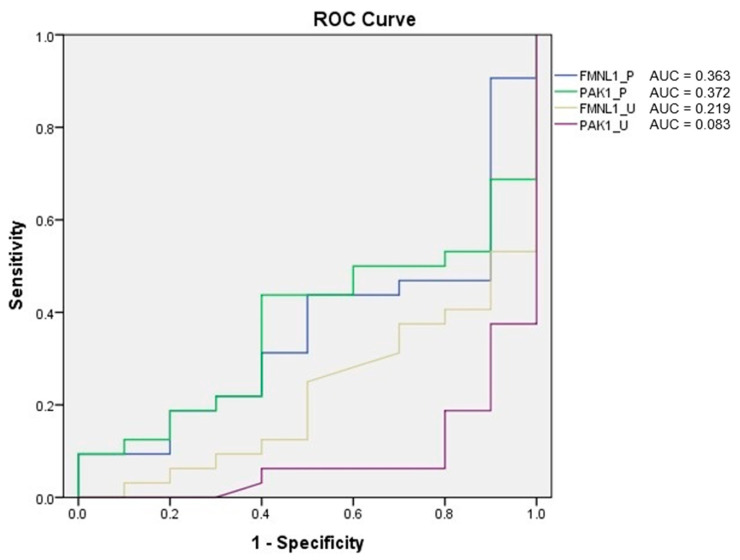
ROC analysis of protein tests.

ROC analysis of the tests was performed, and AUC was calculated according to the ROC curves. As a result of the evaluation, it was determined that the diagnostic value of the tests was low (Figure 12).

**Figure 13:**
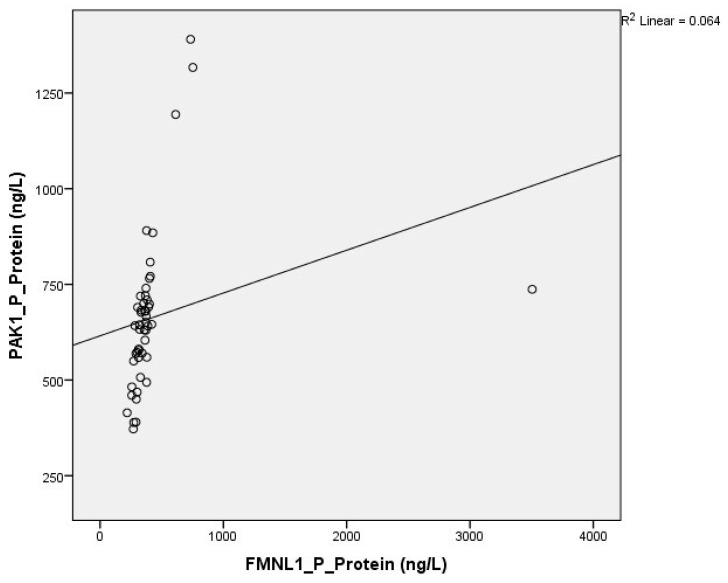
Correlation of plasma protein expressions.

**Figure 14:**
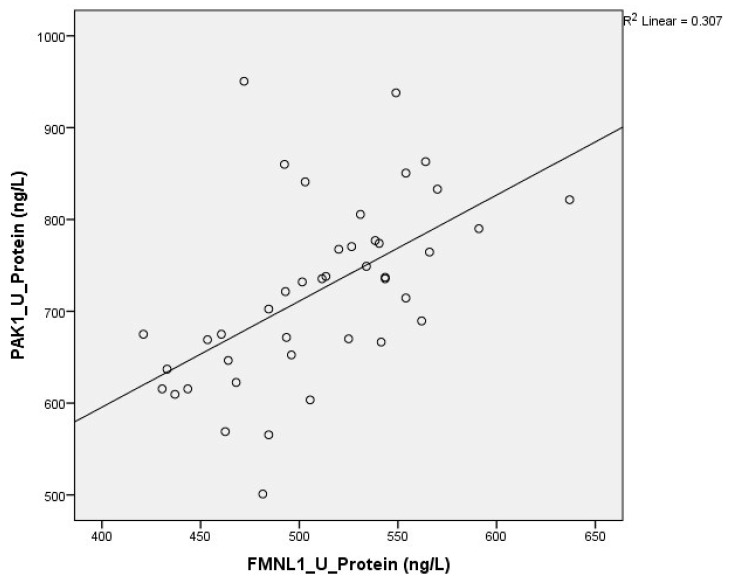
Correlation of urine protein expressions.

Correlation between tests was determined with the Spearman test. A correlation was found between plasma protein FMNL1 levels and urine protein PAK1 levels (*p* = 0.000, r = 0.779) (Figure 13 and Figure 14).

## 4. Discussion

PCa is a type of cancer that has been the subject of much research due to its incidence, development of metastases, and related reduction in quality of life. The fact that the methods used routinely for the diagnosis of the disease, the follow-up of the treatment effectiveness, and the evaluation of the risk of metastasis do not provide sufficient information, which keeps the search for both biochemical and molecular markers on the agenda.

Cancer metastasis includes steps of EMT, invasion, intravasation, extravasation, and metastatic colony formation. Decreased expression and loss of function of proteins that provide cell connections and increased expression of proteins that disrupt the ECM structure, deconstruct the cytoskeleton, and lead to the spread of the tumor. Actin microfilaments, one of the basic structural elements of the cytoskeleton, determine the shape and movement of the cell by providing mechanical support to the cell. As a result of the structural change in the actin skeleton, the formation of cell extensions, an increase in cell movement, and a decrease in cell–matrix connections (adhesion plates) and cell–cell junction complexes make it easier for the cell to gain invasive capacity.

Actin-based processes such as cell division, membrane trafficking, migration, morphogenesis, and filopodium formation have been increasingly associated with tumor invasion and metastasis. Formins, known as regulators of actin dynamics, increase cell movement by stimulating actin polymerization and play a role in tumor invasion and metastasis. FMNL1, which is located in the cytoplasm together with actin, is thought to play a role in basic actin-related processes such as nucleation, elongation, polymerization, depolymerization, and packaging, but its function has not been fully elucidated.

It has been determined that the expression of FMNL1 is increased in many cancer types of hematopoietic origin, such as myeloid leukemia, lymphoid leukemia, and non-Hodgkin lymphoma, but its expression in solid tumors is a subject that has just begun to be studied [18,19]. In the studies published to date, no study has been found in blood and other body fluids. Our study is the first one on the efficacy of this molecule in PCa, as well as the only study investigating its expression in plasma and urine.

FMNL1 was first isolated in lymphoid tumors by Favaro et al. in 2003 [3]. In the subsequent studies of the same team, high expression was detected in T-cell non-Hodgkin lymphomas by both Western blot and immunohistochemistry methods, and it was stated that it is also effective in the proliferation and migration of leukemia cells [18,19]. In the study of Kilpinen and his team in 2008, the FMNL1 mRNA expression profile was obtained in both healthy and tumor samples of 18 different tissue types, and its expression was observed, albeit slightly, in both healthy and tumor tissues of the prostate [20]. In 2011, in the study where FMNL1 was shown for the first time in epithelial cancers, its expressions were also found in Jurkat, HeLa, and pancreatic cancer cell lines. These findings lead to the idea that there is high FMNL1 expression in solid tumors with high metastasis potential [21].

As the first study to report FMNL1 protein expression in cancer types other than hematological cancers, the study conducted in 2014, in which 28 different cancer tissues were examined, stands out. In the examination of many tissues, including PCa, by the immunohistochemistry method, no expression was found in epithelial cells, but high expression was observed in smooth muscle cells [22]. Since the cells shed outside the tissue are usually of epithelial tissue origin, the fact that we encountered low levels in plasma and urine in our patient group is consistent with the findings of this study.

In the study by Yi Yang et al., FMNL1 mRNA expression and protein levels were examined in 117 non-small-cell lung cancer (NSCLC) tissues, 37 in NSCLC bone metastasis tissues, and 70 in normal lung tissues, and high levels have been detected. High FMNL1 levels have been associated with a poor prognosis of the disease. In addition, they showed that silencing FMNL1 inhibited cell migration and slowed down bone metastasis, and they stated that FMNL1 plays an important role in regulating bone metastases in NSCLC [23]. Although we thought that close results could be seen with this study since PCa is a tumor type with bone metastases mostly, we could not obtain a result that could be evaluated statistically in this context, since the number of metastatic patients was very low in our study.

In the study of Higa et al., FMNL1 expression was examined by immunohistochemistry in tissue samples of 217 cases of glioblastoma, and as a result of the study, it was explained that high FMNL1 levels were an indicator of poor prognosis and low survival, and that FMNL1 silencing blocked cell migration and invasion in glioblastoma by suppressing actin polymerization. The significant association of FMNL1 with lamellipod, focal adhesion, invadopod, metastasis, and migration-related gene sets further strengthens the idea that this gene affects the aggressive phenotype of the tumor [24].

As a result of the immunohistochemistry and mRNA expression study performed in 306 clear cell renal cell carcinoma tissues, it was observed that FMNL1 was highly expressed in clear cell renal cell carcinoma and increased prometastatic activity. It has been stated that high FMNL1 expression is associated with high tumor grade, tumor metastasis, and poor prognosis by increasing cell migration and invasion [25].

In our study, no statistical difference was found between FMNL1 mRNA expression levels of PCa patients and controls in both plasma and urine samples. Likewise, no statistical significance was found in protein levels (*p* > 0.05). No correlation was found between PSA and other clinical characteristics of the patients and plasma and urine FMNL1 levels.

It is known that PAK family members have high expression and activation in various cancer types. Among PAK isoforms, group I PAKs (especially PAK1 and PAK2) are the best characterized and most deregulated in cancers [26,27,28].

Studies on PAK1 have been conducted mostly in gastrointestinal tract tumors, as well as in ovarian, head and neck, pancreatic, and prostate cancers.

A gradual increase in PAK1 protein and mRNA levels in gastroesophageal tumor tissues was observed along the progression towards normal epithelium, atypical hyperplasia, and adenocarcinoma, and PAK1 overexpression was associated with lymph node metastasis, advanced tumor stage, large tumor size, positive surgical margins, and poor survival [29].

In studies conducted on gastrointestinal system cancers, it has been stated that PAK1 expression and activity are associated with the aggressive behavior of the tumor. Activation of PAK1 increases cell motility and invasion by preventing the formation of focal adhesion complexes, while its silencing reduces cell migration and invasion [30,31,32].

In the study conducted by Park J and his team on head and neck carcinoma cells, it was stated that PAK1 plays a role in tumor cell migration and invasion, and its overexpression correlates with the aggressive behavior of the tumor cell and low survival [33].

In the expression study conducted by Han and his team in primary and metastatic pancreatic cancer tissues, lower PAK1 levels were detected in metastatic tissues than in primary tissues. PAK1 levels in primary tumor tissues were also found to be associated with a good differentiation degree and longer survival, contrary to other studies in the literature. As a result of the study, it was stated that the effect of PAK1 on pancreatic cancer metastasis may be low, and these findings suggest that it does not have the same effect in every tissue and its cellular functions may vary [34].

It is known that PAK4 and PAK6 from group II PAKs (PAK4, 5, 6) are highly expressed in PCa [35,36]. Therefore, although present in prostate tissue, group I PAKs (PAK1, 2, 3) have not been studied much in PCa.

In the first study on the role of PAK1 in PCa, PAK1 levels were compared with PAK6 in tissues and cell lines. In the study published in 2013, PAK1 expression was observed to be much higher in cell lines with high invasive/metastatic potential than in early-stage tumor cell lines, while PAK6 expression was detected at similar levels in all cell lines. While PAK1 protein levels were at their lowest values in normal and benign hyperplasia tissues, they were found to be much higher in tumor and metastatic tissues [37].

In a study conducted with 73 PCa and 40 benign prostate hyperplasia patients, PAK1 levels were found to be correlated with PSA values, and PAK1 mRNA levels were detected to be highest in metastatic cell lines, then in early-stage PCa cell lines, and the lowest in normal prostate epithelial cell lines. It has been shown that proliferation, migration, and invasion are slowed down by silencing PAK1 gene expression [38].

In our study, no statistical difference was found in plasma samples between PAK1 mRNA expression levels of PCa patients and controls (*p* > 0.05).

While there was no significant difference in protein levels in plasma samples (*p* > 0.05), it was found to be lower in urine samples in patients than in the control group (*p* = 0.00). No correlation was found between PSA and other clinical characteristics of the patients and plasma and urine FMNL1 levels.

In order for tumor cells to be detected in the circulation, the cells must separate from each other, adhere to ECM components, degrade the ECM, and move out of the vessel. In local stage cancers, it is difficult to detect cellular elements of the tumor in the circulation or other body fluids, since the tumor cells are not yet in the spreading stage. In studies conducted on tumor tissue, tumor cells and their components can be more easily detected both qualitatively and quantitatively, regardless of the stage of the disease.

While we think that FMNL1 and PAK1 will show high expression in patients with PCa, in line with the information in the literature, we think that our results are in this direction since we studied outside the tissue and mostly in early-stage patients. Our study is the first study in which both the gene and protein product of FMNL1 and PAK1 were detected and compared in circulation and urine, and it is important because it shows that both marker molecules have low expression levels in early-stage PCa.

We consider it scientifically appropriate to extend this preliminary study by including tissue samples from patients with more advanced stages, especially those with metastatic disease.

## 5. Conclusions

In this study on prostate cancer, we investigated FMNL1 and PAK1 in prostate cancer patients. Although we did not observe statistically significant and highly impactful results, this research represents one of the first efforts in this specific area. We believe that our findings contribute to the literature by providing a foundation for future studies and hold promise for advancing understanding in this field. Further research with larger cohorts and extended methodologies is warranted to validate and expand upon these preliminary observations.

## Figures and Tables

**Table 1:** Characteristics of patients.

		Age		Stage		
		<65	≥65	Local	Local Advanced	Metastatic
N	60	21	39	38	18	4
%	100	35	65	63.3	30	6.7

**Table 2:** cDNA synthesis reaction components.

Reaction Components	Volume for 1 Sample
Rnase-free Water	10.5 μL
SCRIPT RT Reaction Buffer	4 μL
10 mM Deoxynucleotide Mixture	1 μL
DTT Stock Solution	1 μL
RNase Inhibitor	1 μL
SCRIPT Reverse Transkriptase	0.5 μL
RNA Product	2 μL

**Table 3:** Primers for the studied genes.

Gene	Forward Primer F (5′-3′)	Reverse Primer R (5′-3′)
FMNL1	AACTTCCGTGTCTTCCTGCAA	CTTGTCACTCTCGGTGAGCC
PAK1	TTGTGAAATGCTCTCGGCTATG	TCACCATTTTCCCCGACTTC

**Table 4:** Arithmetic mean (x), standard deviation (sd), median (m), minimum (min), and maximum (max) values of mRNA expressions in PCa patients and healthy control groups.

mRNA Expression Levels	Prostate Cancer (*n* = 60) x ± sd m (min, max)	Control (*n* = 20) x ± sd m (min, max)	*p*-Value
Plasma FMNL1	1.33 ± 1.96 0.66 (0.07–13.44)	1.64 ± 1.61 1.44 (0.09–5.78)	0.182
Urine FMNL1	1.93 ± 6.58 0.42 (0.01–48.44)	1.06 ± 0.85 0.83 (0.15–3.55)	0.126
Plasma PAK1	0.70 ± 1.37 0.15 (0.02–5.43)	0.96 ± 1.52 0.25 (0.03–4.85)	0.191
Urine PAK1	1.07 ± 2.16 0.39 (0.01–11.48)	0.93 ± 0.66 0.83 (0.03–2.09)	0.071

**Table 5:** Arithmetic mean (x), standard deviation (sd), median (m), minimum (min), and maximum (max) values of protein levels in PCa patients and healthy control groups.

Protein Levels (ng/L)	Prostate Cancer (*n* = 60) x ± sd m (min, max)	Control (*n* = 20) x ± sd m (min, max)	*p*-Value
Plasma FMNL1	425.9 ± 460.2 357.5 (219.0–3505.0)	389.7 ± 68.4 379.8 (266.5–553.5)	0.077
Urine FMNL1	509.5 ± 47.8 508.5 (421.0–637.0)	520.7 ± 80.3 544.8 (330.0–592.0)	0.068
Plasma PAK1	682.5 ± 504.4 631.8 (242.5–4175.0)	683.3 ± 101.7 658.5 (514.5–846)	0.089
Urine PAK1	614.3 ± 201.1 669.5 (197.5–950.5)	868.8 ± 117.2 893.5 (457.0–1005.0)	0.000

## Data Availability

The original contributions presented in this study are included in this article. Further inquiries can be directed to the corresponding author.

## Abbreviations

The following abbreviations are used in this manuscript:

FMNL1	Formin-like-protein 1
PAK1	p21-activated kinase 1
PCa	Prostate cancer
PSA	Prostate-specific antigen
EMT	Epithelial to mesenchymal transition
Ct	Threshold cycle
ROC	Receiver operating characteristic
AUC	Area under curve
NSCLC	Non-small-cell lung cancer

## References

[1] Dhawan M., Ryan C.J., Ashworth A. (2016). DNA Repair Deficiency Is Common in Advanced Prostate Cancer: New Therapeutic Opportunities. Oncologist.

[2] Higgs H.N. (2005). Formin proteins: A domain-based approach. Trends Biochem. Sci..

[3] Favaro P.M., De Souza Medina S., Traina F., Bassères D.S., Costa F.F., Saad S.T. (2003). Human leukocyte formin: A novel protein expressed in lymphoid malignancies and associated with Akt. Biochem. Biophys. Res. Commun..

[4] Esue O., Harris E.S., Higgs H.N., Wirtz D. (2008). The filamentous actin cross-linking/bundling activity of mammalian formins. J. Mol. Biol..

[5] Harris E.S., Rouiller I., Hanein D., Higgs H.N. (2006). Mechanistic differences in actin bundling activity of two mammalian formins, FRL1 and mDia2. J. Biol. Chem..

[6] Miller M.R., Blystone S.D. (2015). Human Macrophages Utilize the Podosome Formin FMNL1 for Adhesion and Migration. Cellbio.

[7] Chen W.H., Cai M.Y., Zhang J.X., Wang F.W., Tang L.Q., Liao Y.J., Jin X.H., Wang C.Y., Guo L., Jiang Y.G. (2018). FMNL1 mediates nasopharyngeal carcinoma cell aggressiveness by epigenetically upregulating MTA1. Oncogene.

[8] Manser E., Leung T., Salihuddin H., Zhao Z.S., Lim L. (1994). A brain serine/threonine protein kinase activated by Cdc42 and Rac1. Nature.

[9] Zhang S., Han J., Sells M.A., Chernoff J., Knaus U.G., Ulevitch R.J., Bokoch G.M. (1995). Rho family gtpases regulate p38 mitogen-activated protein kinase through the downstream mediator pak1. J. Biol. Chem..

[10] Sells M.A., Knaus U.G., Bagrodia S., Ambrose D.M., Bokoch G.M., Chernoff J. (1997). Human p21-activated kinase (pak1) regulates actin organization in mammalian cells. Curr. Biol..

[11] Liu K.H., Huynh N., Patel O., Shulkes A., Baldwin G., He H. (2013). P21-activated kinase 1 promotes colorectal cancer survival by up-regulation of hypoxia-inducible factor-1alpha. Cancer Lett..

[12] Alavi A., Hood J.D., Frausto R., Stupack D.G., Cheresh D.A. (2003). Role of raf in vascular protection from distinct apoptotic stimuli. Science.

[13] Yao D., Li C., Rajoka M.S.R., He Z., Huang J., Wang J., Zhang J. (2020). P21-Activated Kinase 1: Emerging biological functions and potential therapeutic targets in Cancer. Theranostics.

[14] Zhu Y., Liu H., Xu L., An H., Liu W., Liu Y., Lin Z., Xu J. (2015). P21-activated kinase 1 determines stem-like phenotype and sunitinib resistance via nf-kappab/il-6 activation in renal cell carcinoma. Cell Death Dis..

[15] He H., Huynh N., Liu K.H., Malcontenti-Wilson C., Zhu J., Christophi C., Shulkes A., Baldwin G.S. (2012). P-21 activated kinase 1 knockdown inhibits beta-catenin signalling and blocks colorectal cancer growth. Cancer Lett..

[16] Kim E., Youn H., Kwon T., Son B., Kang J., Yang H.J., Seong K.M., Kim W., Youn B. (2014). Pak1 tyrosine phosphorylation is required to induce epithelial-mesenchymal transition and radioresistance in lung cancer cells. Cancer Res..

[17] Williams E.D., Gao D., Redfern A., Thompson E.W. (2019). Controversies around epithelial-mesenchymal plasticity in cancer metastasis. Nat. Rev. Cancer.

[18] Favaro P.M., Traina F., Vassallo J., Brousset P., Delsol G., Costa F.F., Saad S.T. (2006). High expression of FMNL1 protein in T non-Hodgkin’s lymphomas. Leuk. Res..

[19] Favaro P., Traina F., Machado-Neto J.A., Lazarini M., Lopes M.R., Pereira J.K., Costa F.F., Infante E., Ridley A.J., Saad S.T. (2013). FMNL1 promotes proliferation and migration of leukemia cells. J. Leukoc. Biol..

[20] Kilpinen S., Autio R., Ojala K., Iljin K., Bucher E., Sara H., Pisto T., Saarela M., Skotheim R.I., Björkman M. (2008). Systematic bioinformatic analysis of expression levels of 17,330 human genes across 9783 samples from 175 types of healthy and pathological tissues. Genome Biol..

[21] Colón-Franco J.M., Gomez T.S., Billadeau D.D. (2011). Dynamic remodeling of the actin cytoskeleton by FMNL1γ is required for structural maintenance of the Golgi complex. J. Cell Sci..

[22] Gardberg M., Heuser V.D., Iljin K., Kampf C., Uhlen M., Carpén O. (2014). Characterization of Leukocyte Formin FMNL1 Expression in Human Tissues. J. Histochem. Cytochem..

[23] Yang X.Y., Liao J.J., Xue W.R. (2019). FMNL1 down-regulation suppresses bone metastasis through reducing TGF-β1 expression in non-small cell lung cancer (NSCLC). Biomed. Pharmacother..

[24] Higa N., Shinsato Y., Kamil M., Hirano T., Takajo T., Shimokawa M., Minami K., Yamamoto M., Kawahara K., Yonezawa H. (2019). Formin-like 1 (FMNL1) Is Associated with Glioblastoma Multiforme Mesenchymal Subtype and Independently Predicts Poor Prognosis. Int. J. Mol. Sci..

[25] Zhang M.F., Li Q.L., Yang Y.F., Cao Y., Zhang C.Z. (2020). FMNL1 Exhibits Pro-Metastatic Activity via CXCR2 in Clear Cell Renal Cell Carcinoma. Front. Oncol..

[26] Kumar R., Gururaj A.E., Barnes C.J. (2006). p21-activated kinases in cancer. Nat. Rev. Cancer.

[27] Kichina J.V., Goc A., Al-Husein B., Somanath P.R., Kandel E.S. (2010). PAK1 as a therapeutic target. Expert Opin. Ther. Targets.

[28] Eswaran J., Lee W.H., Debreczeni J.E., Filippakopoulos P., Turnbull A., Fedorov O., Deacon S.W., Peterson J.R., Knapp S. (2007). Crystal Structures of the p21-activated kinases PAK4, PAK5, and PAK6 reveal catalytic domain plasticity of active group II PAKs. Structure.

[29] Li Z., Zou X., Xie L., Dong H., Chen Y., Liu Q., Wu X., Zhou D., Tan D., Zhang H. (2013). Prognostic importance and therapeutic implications of PAK1, a drugable protein kinase, in gastroesophageal junction adenocarcinoma. PLoS ONE.

[30] Song B., Wang W., Zheng Y., Yang J., Xu Z. (2015). P21-activated kinase 1 and 4 were associated with colorectal cancer metastasis and infiltration. J. Surg. Res..

[31] Li L.H., Zheng M.H., Luo Q., Ye Q., Feng B., Lu A.G., Wang M.L., Chen X.H., Su L.P., Liu B.Y. (2010). P21-activated protein kinase 1 induces colorectal cancer metastasis involving ERK activation and phosphorylation of FAK at Ser-910. Int. J. Oncol..

[32] Liu F., Li X., Wang C., Cai X., Du Z., Xu H., Li F. (2009). Downregulation of p21-activated kinase-1 inhibits the growth of gastric cancer cells involving cyclin B1. Int. J. Cancer.

[33] Park J., Kim J.M., Park J.K., Huang S., Kwak S.Y., Ryu K.A., Kong G., Park J., Koo B.S. (2015). Association of p21-activated kinase-1 activity with aggressive tumor behavior and poor prognosis of head and neck cancer. Head Neck.

[34] Han J., Wang F., Yuan S.Q., Guo Y., Zeng Z.L., Li L.R., Yang J., Wang D.S., Liu M.Y., Zhao H. (2014). Reduced expression of p21-activated protein kinase 1 correlates with poor histological differentiation in pancreatic cancer. BMC Cancer.

[35] Schrantz N., Da Silva Correia J., Fowler B., Ge Q., Sun Z., Bokoch G.M. (2004). Mechanism of p21-activated kinase 6-mediated inhibition of androgen receptor signaling. J. Biol. Chem..

[36] Yang F., Li X., Sharma M., Zarnegar M., Lim B., Sun Z. (2001). Androgen receptor specifically interacts with a novel p21-activated kinase, PAK6. J. Biol. Chem..

[37] Goc A., Al-Azayzih A., Abdalla M., Al-Husein B., Kavuri S., Lee J., Moses K., Somanath P.R. (2013). P21 activated kinase-1 (Pak1) promotes prostate tumor growth and microinvasion via inhibition of transforming growth factor β expression and enhanced matrix metalloproteinase 9 secretion. J. Biol. Chem..

[38] Wang Z., Jia G., Li Y., Liu J., Luo J., Zhang J., Xu G., Chen G. (2017). Clinicopathological signature of p21-activated kinase 1 in prostate cancer and its regulation of proliferation and autophagy via the mTOR signaling pathway. Oncotarget.

